# Fructose-1,6-Bisphosphate Reduces Chronic Constriction Injury Neuropathic Pain in Mice by Targeting Dorsal Root Ganglia Nociceptive Neuron Activation

**DOI:** 10.3390/ph18050660

**Published:** 2025-04-30

**Authors:** Amanda Martins Dionisio, Paula de Azevedo Oliveira Milanez, Ana Carla Zarpelon-Schutz, Sandra Satie Mizokami, Mariana Marques Bertozzi, Kelly Megumi Yaekashi, Doumit Camilios-Neto, Sergio Marques Borghi, Rubia Casagrande, Waldiceu A. Verri

**Affiliations:** 1 Laboratory of Pain, Inflammation, Neuropathy, and Cancer, Department of Immunology, Parasitology and General Pathology, Center of Biological Sciences, Londrina State University, Londrina 86057-970, PR, Brazil; 2 Department of Biochemistry and Biotechnology, Centre of Exact Sciences, Londrina State University, Londrina 86057-970, PR, Brazil; 3 Department of Pharmaceutical Sciences, Center of Health Science, Londrina State University, Londrina 86038-440, PR, Brazil

**Keywords:** fructose-1,6-bisphosphate, adenosine A1 receptor, adenosine A2A receptor, neuropathic pain, NO/cGMP/PKG/K^+^ATP signaling pathway, mouse

## Abstract

**Background/Objectives:** Fructose-1,6-bisphosphate (FBP) is an intermediate product of the glycolytic pathway with analgesic effect in acute inflammatory pain model via the production of adenosine. However, whether FBP is active in neuropathic pain is unknown. Therefore, we reason that it would be suitable to investigate the analgesic effect and mechanism of action of FBP in a model of chronic constriction injury (CCI) of sciatic nerve-induced neuropathic pain in mice. **Methods:** After CCI induction, mice received FBP, adenosine, A1 and/or A2A receptor antagonists, and/or inhibitors of the nitric oxide (NO)/cyclic guanosine monophosphate (cGMP)/protein kinase G (PKG)/ATP sensitive K channels (KATP) signaling pathway. **Results:** FBP (up to 85%) and adenosine (up to 84%) inhibited the mechanical hyperalgesia (electronic aesthesiometer) induced by CCI with similar profiles. FBP analgesia was dependent on adenosine because adenosine A1 and A2A receptors antagonists diminished FPB activity (100% and 79%, respectively). FBP analgesia was also dependent on activating the NO/cGMP/PKG/KATP signaling pathway. Furthermore, FBP treatment increased the production of NO in cultured dorsal root ganglia (DRG) neurons (100% increase), whereas neuronal nitric oxide synthase (nNOS) inhibition decreased (up to 70%) the analgesic effect of FBP. We also observed that FBP reduced the calcium levels of transient receptor potential ankyrin 1 (TRPA1)^+^ DRG neurons (85%) and paw-flinching triggered by TRPA1 activation (38%). **Conclusions**: FBP reduced neuropathic pain by reducing DRG neuron activation. The mechanisms involved the activation of adenosine A1 and A2A receptors to trigger the analgesic NO/cGMP/PKG/KATP signaling pathway and reducing TRPA1^+^ DRG neuron activity.

## 1. Introduction

Pain sensation occurs when specialized primary afferent neurons localized in the peripheral nervous system (PNS), named nociceptors, sense danger, recognizing intense stimuli as thermal, mechanical, or chemical, leading to the activation of the transient receptor potential (TRP) channel and modulation of other channels, such as the sodium channels (Nav1.7, Nav1.8, and Nav1.9) [1,2,3]. TRP ankyrin 1 (TRPA1) channel have a very important role in pain. TRPA1 is expressed by nociceptive dorsal root ganglia (DRG) neurons [1]. TRPA1 can be activated by electrophilic agents, such as allyl isothiocyanate (AITC), which is a pungent component present in wasabi and mustard oil, and can also be activated by classic nociceptive stimulation, such as formalin [2,3]. Thus, TRPA1 is important for the transduction of irritants and endogenous agents to depolarize nociceptors, inducing pain and mechanical hyperalgesia [4].

The sensation of pain can be classified, according to the International Association for the Study of Pain (IASP) (2017), into nociceptive, nociplastic, and neuropathic pain [5,6]. Focusing on neuropathic pain, it is described as “pain induced by an injury or disease of the somatosensory nervous system” [5,7] by the IASP. The symptoms associated with neuropathic pain can affect quality of life, both physically and psychologically. This is a global health issue, affecting approximately 7 to 10% of the general population and 24% of individuals with various types of chronic pain [8,9,10,11]. Pharmacological approaches typically prescribed to treat neuropathic pain can be briefly summarized as belonging to three lines in which gabapentin and pregabalin (side-effects include sleepiness, dizziness, dry mouth, and vision impairment), noradrenalin and serotonin reuptake inhibitors (side-effects include nausea, vomiting, fatigue, and dizziness), and tricyclic antidepressants (side-effects include blurred vision, urinary retention, constipation, and gain of weight) represent the first line options. The second-line treatments include patches containing capsaicin and lidocaine (side-effects include temporary burning sensation and skin redness). The third line encompasses tramadol and strong opioids [12], which can lead to chemical addiction if used for prolonged periods, adding another problem for patients and society [13,14,15]).

Fructose-1,6-biphosphate (FBP) is an endogenous intermediate of the glycolytic pathway [16,17]. It is described in the literature that FBP has a protective action in different cell types and in tissues during ischemia and hypoxia, demonstrating immunological, neuroprotective, and anti-inflammatory effects [18,19,20], by modulating varied signaling pathways [13]. Also, FBP administration induces analgesia that depends on the activation of, at least, the adenosine A1 receptor. Indeed, FBP treatment induces an increase in adenosine (ADO) blood levels, and FBP and ADO mimic each other’s analgesic activity [21]. ADO, a purine nucleoside, is continuously formed, both intracellularly and extracellularly, under physiological conditions [22]. ADO promotes analgesia in diabetic neuropathy and spinal nerve ligation neuropathic pain in rats [23,24] and in humans with neuropathic pain [25,26,27,28]. ADO receptor A1 is expressed in neurons located in the brain [29,30] and also in the peripheral primary afferent nociceptor neurons, whose cellular bodies are in the DRG [31]. As mentioned earlier, A1 receptor agonists have antinociceptive effects in several pain models, such as formalin test [32,33], chronic constriction injury (CCI) induced-neuropathic pain [34,35], nerve injury neuropathy, and mono-arthritis [36,37]. The ADO A2A receptors are expressed by almost all cells that participate in the immune response, such as macrophages, monocytes, dendritic cells, mast cells, neutrophils, eosinophils, lymphocytes, glial cells (astrocytes and microglia) endothelial cells, and epithelial cells [38,39,40]. However, the effect of the A2A receptor in the modulation of inflammation and pain remains controversial. In addition, in the neuropathic pain model, treatment with A2A receptor agonists (ATL313 and CGS21680) reduces allodynia induced by CCI of the sciatic nerve [41]. In turn, ADO reduces neuropathic pain [42]. The activation of the NO/cGMP/PKG/ATP-sensitive potassium channel signaling pathway can induce an analgesic effect in neuropathic pain models [43,44,45]. Interestingly, this is the same signaling pathway used by opioids and some NSAIDs and analgesics to generate their analgesic mechanisms, thus suggesting that this is an important analgesia pathway [46].

Despite the evidence that FBP can diminish inflammatory pain by mechanisms that suggest potential analgesic activity in neuropathic pain, to our knowledge, it has not been investigated whether FBP can reduce neuropathic pain. Therefore, we reason that it would be a valuable addition to provide a proof-of-concept on whether FBP is analgesic in neuropathic pain and offer further insight into its analgesic mechanisms. In the present study, we focused on the activity of FBP in the peripheral nociceptor neurons using the CCI model.

## 2. Results

### 2.1. FBP Reduces Mechanical Hyperalgesia Induced by CCI in a Dose-Dependent Manner

**Figure 1:**
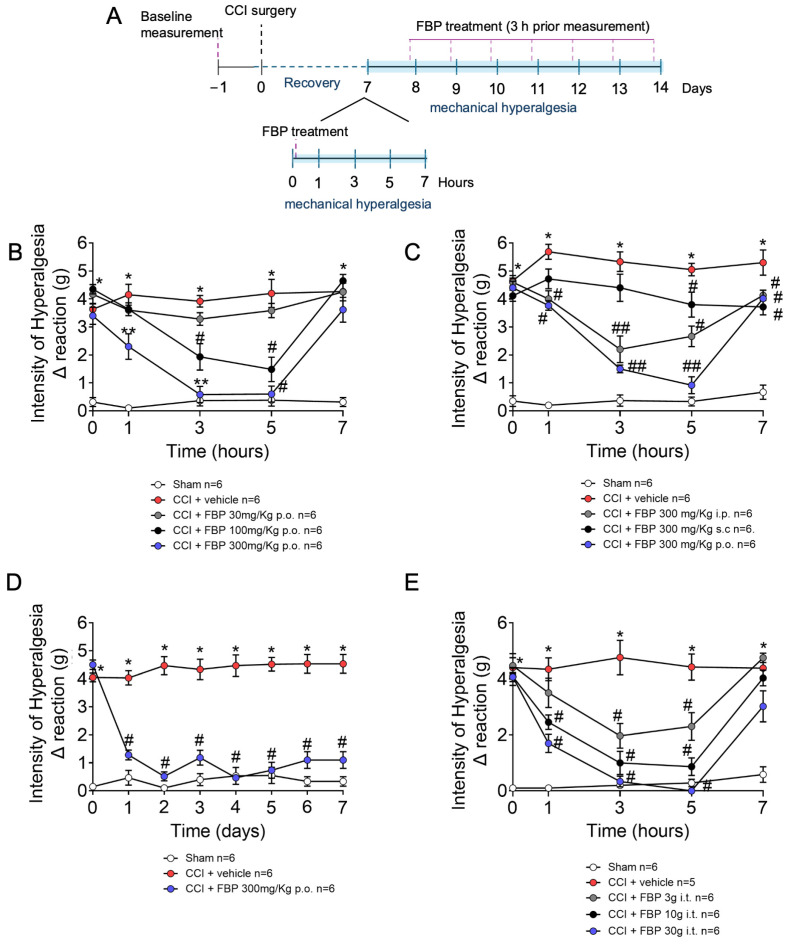
FBP reduces CCI-induced mechanical hyperalgesia. (**A**) Scheme of experimental design for this figure; (**B**) FBP (10–300 mg/kg) dose–response effect in CCI-induced mechanical hyperalgesia 1–7 h after treatment; (**C**) Effect of different routes of FBP administration on CCI-induced mechanical hyperalgesia 1–7 h after treatment; (**D**) Chronic FBP treatment for 7 days on CCI-induced mechanical hyperalgesia; and (**E**) Effects of intrathecal administration of FBP on CCI-induced mechanical hyperalgesia 1–7 h after treatment. *n* = 6 mice per group experiment. * *p* < 0.05 vs. Sham group; # *p* < 0.05 vs. CCI + vehicle group; ** *p* < 0.05 vs. CCI + vehicle and CCI + FBP 100 mg/kg groups; ## *p* < 0.05 vs. CCI + FBP 300 mg/kg s.c. (Two-way ANOVA followed by Tukey’s post-test).

This study initiated by inducing CCI neuropathy and at the seventh day post-surgery performing a per-oral (p.o.) dose-response for FBP (30–300 mg/kg), followed by mechanical hyperalgesia measurement 1–7 h after treatment (Figure 1A). There was a significant increase in mechanical hyperalgesia in CCI mice compared with the sham group. FBP treatment reduced in a dose-dependent manner CCI mechanical hyperalgesia (Figure 1B). The dose of 300 mg/kg significantly reduced the mechanical hyperalgesia from the the first to the fifth hour, while the 100 mg/kg dose reduced pain 3 and 5 h after the p.o. treatment, with lessened activity compared with the 300 mg/kg dose. Therefore, the dose of 300 mg/kg was chosen for the next sets of experiments using p.o. treatment. To further evaluate and compare the efficacy of the effects of different routes of administration, mice were treated with 300 mg/kg of FPB by p.o., intraperitoneal (i.p.), and subcutaneous (s.c) vias. FBP p.o. and i.p. reduced the mechanical hyperalgesia from the first hour up to seven hours after administration (Figure 1C). The s.c. administration of FPB reduced the mechanical hyperalgesia 5 and 7 h after treatment. There was a significant difference between p.o. and s.c. administration at 3 and 5 h, and between i.p. and s.c. at 3 h after FBP treatment. Next, we evaluated the daily FPB treatment between 7 and 14 days after CCI surgery. The p.o. route was selected based on the data in Figure 1C. For that, mice were treated with 300 mg/kg p.o. daily, and the mechanical hyperalgesia was measured for 7 days. The FPB daily treatment reduced mechanical hyperalgesia at all time points, demonstrating its applicability for prolonged treatment (Figure 1D). The intrathecal (i.t.) route of administration allows for verifying if a drug could present effects in the spinal cord and dorsal root ganglia; therefore, we also tested the activity of FBP treatment (3–30 µg per mouse) by the i.t. route and assessed mechanical hyperalgesia from 1 to 7 h after treatment. All doses reduced CCI-induced mechanical hyperalgesia when compared with the vehicle-treated group (Figure 1E). These data highlight that treatment with FBP by the p.o. route achieves a better analgesic effect, replicated by direct spinal delivery using i.t. treatment.

### 2.2. ADO Reduces Mechanical Hyperalgesia Induced by CCI in a Dose-Dependent Manner

**Figure 2:**
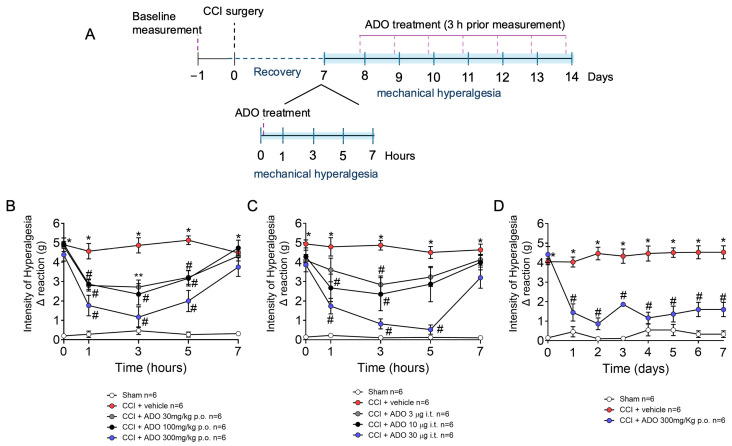
ADO reduces CCI-induced mechanical hyperalgesia. (**A**) Schematic representation of experimental design in this figure. (**B**) ADO (10–300 mg/kg) dose–response on CCI-induced mechanical hyperalgesia 1–7 h after treatment; (**C**) Chronic ADO treatment for 7 days on CCI-induced mechanical hyperalgesia; and (**D**) Effects of intrathecal administration of ADO on CCI-induced mechanical hyperalgesia 1–7 h after treatment. *n* = 6 mice per group per experiment. * *p* < 0.05 vs. sham group; # *p* < 0.05 vs. CCI + vehicle group; ** *p* < 0.05 vs. vehicle group and CCI + ADO 300 mg/kg group (Two-way ANOVA followed by Tukey’s post-test).

Considering that FBP activities are dependent on ADO under varied conditions, we tested whether ADO would mimic the activity of FBP (Figure 2A). Seven days after CCI surgery, mechanical hyperalgesia was assessed before and after ADO (30–300 mg/kg) p.o. treatment. ADO treatment reduced mechanical hyperalgesia in a dose–response manner. The doses of 30, 100, and 300 mg/kg reduced mechanical hyperalgesia when compared with the CCI group treated with vehicle from 1 to 5, and maximal activity was obtained with 300 mg/kg of ADO (Figure 2B). Next, the effect of prolonged treatment with ADO for 7 days was then investigated with a dose of 300 mg/kg. ADO reduced mechanical hyperalgesia at all time points evaluated when compared with the CCI group treated with the vehicle between 7 and 14 days post-surgery, demonstrating its potential for prolonged treatment (Figure 2D). Lastly, the effect of i.t. administration of ADO (3, 10, and 30 µg) on CCI-induced mechanical hyperalgesia was investigated. A reduction in CCI mechanical hyperalgesia was observed with the doses of 10 and 30 µg, reaching significant differences from 1 to 5 h when compared with the CCI group treated with vehicle (Figure 2C). These results reveal that ADO possesses analgesic effects in CCI-induced mechanical hyperalgesia similar to FBP.

### 2.3. Analgesic Effect of FBP on CCI-Induced Mechanical Hyperalgesia Is Dependent on Adenosine A1 and A2A Receptors

**Figure 3:**
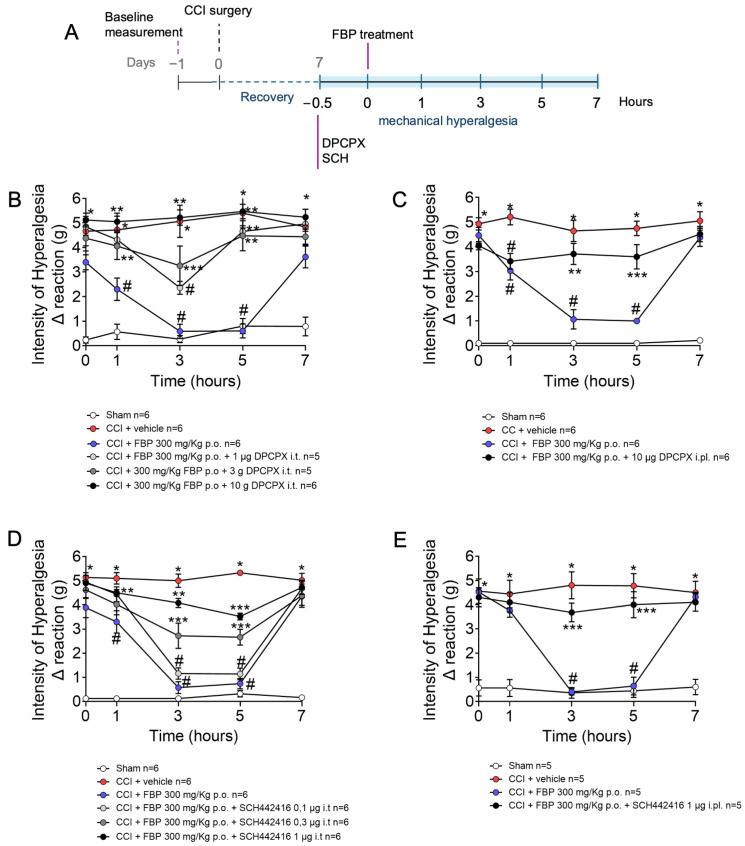
The analgesic effect of FBP on CCI-induced mechanical hyperalgesia is dependent on adenosine A1 and A2 receptors. (**A**) Schematic representation of experimental design in this figure; (**B**) Treatment with DPCPX (A1 receptor antagonist) via i.t. route; (**C**) Treatment with DPCPX (A1 receptor antagonist) via i.pl. route; (**D**) Treatment with SCH442416 (A2A receptor antagonist) via i.t. route; (**E**) Treatment with SCH442416 (A2A receptor antagonist) via i.pl. route. *n* = 5–6 mice per group per experiment. * *p* < 0.05 CCI vs. sham group; # *p* < 0.05 vs. CCI + vehicle group; ** *p* < 0.05 vs. CCI + FBP 300 mg/kg group. *** *p* < 0.05 vs. vehicle group and CCI + FBP 300 mg/kg group (Two-way ANOVA followed by Tukey’s post-test).

To verify whether the FBP analgesic effect is dependent on adenosine receptors, the antagonists of the adenosine A1 and A2A receptors (DPCPX and SCH442416, respectively) were tested (Figure 3A). We opted to use i.t. and intraplantar (i.pl., subcutaneous in the paw) treatment with those antagonists to focus on the contribution of spinal cord and dorsal root ganglia (DRG), as well as peripheral A1 and A2A receptors to the FBP analgesic activity. At the 7th day post-CCI-surgery, DPCPX and SCH442416 were administered via i.t. (1–10 µg and 0.1–1 µg, respectively) or i.pl. (10 µg and 1 µg, respectively) routes. Thirty minutes after antagonists administrations, mice were treated with FBP, and after an additional 1 h, mechanical hyperalgesia was assessed from 1 to 7 h. The i.t. doses of 3 and 10 µg and i.pl. dose of 10 µg of DPCPX abolished the analgesic effect of FBP treatment 1–5 h after the treatment (Figure 3B,C). The analgesia promoted by FBP (1–3 h after administration) was also significantly inhibited by i.t. administration of SCH442416 at doses of 0.3 and 1 µg (Figure 3D). SCH442416 i.pl. treatment (1 µg) equally inhibited the analgesic effect of FBP at 3 and 5 h after (Figure 3E). These results demonstrate that FBP exerts its analgesic effect through A1 and A2A receptors in peripheral and central mechanisms. These results resemble the teleantagonism concept in which primary afferent nociceptor neurons are cells linking the peripheral nervous system (PNS) and central nervous system (CNS), and these neurons can be inhibited by treatments in the paw and by intraganglionic or i.t. administration. Thus, the primary afferent nociceptor neurons can be targeted at the full extent of their anatomic distribution [47].

### 2.4. Analgesic Effect of ADO on CCI-Induced Mechanical Hyperalgesia Is Dependent on Adenosine A1 and A2A Receptors

**Figure 4:**
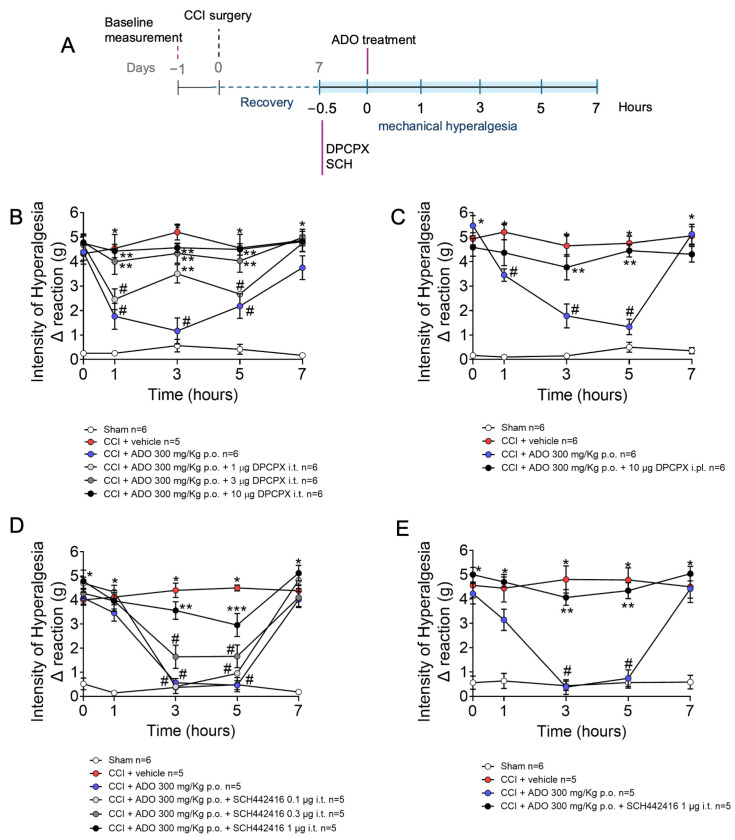
ADO analgesic effect in CCI–induced mechanical hyperalgesia is dependent on adenosine A1 and A2 receptors. (**A**) Schematic of experimental design for this figure; (**B**) Treatment with DPCPX (A1 receptor antagonist) via i.t.; (**C**) Treatment with DPCPX (A1 receptor antagonist) via i.pl.; (**D**) Treatment with SCH442416 (A2A receptor antagonist) via i.t.; (**E**) Treatment with (A2A receptor antagonist) via i.pl. *n* = 5–6 mice per group per experiment. * *p* < 0.05 CCI vs. sham group; # *p* < 0.05 vs. CCI + vehicle group; ** *p* < 0.05 vs. CCI + ADO 300 mg/kg group, *** *p* < 0.05 vs. CCI + vehicle group and ADO 300 mg/kg group (Two-way ANOVA followed by Tukey’s post-test).

We next examined whether the FBP results would be mirrored by ADO, which would further confirm the A1 and A2A receptor mechanism observed. Mice were treated with DPCPX or SCH442416 via i.t. (1–10 µg and 0.1–1 µg, respectively) and i.pl. (10 µg and 1 µg, respectively) routes at the 7th day after CCI surgery. After thirty minutes, mice were treated p.o. with FBP, and mechanical hyperalgesia measured from 1 to 7 h after FBP treatment (Figure 4A). The i.t. doses of 3 and 10 µg of DPCPX abolished the ADO analgesic effect (1–5 h) (Figure 4B). The administration of 10 µg of DPCPX via the i.pl. route also inhibited the analgesic effect of FBP in the third and fifth hours (Figure 4C). The ADO A2A receptor antagonist also reduced the analgesic effect of FBP in both routes of administration. The i.t. dose of 1 µg of SCH442416 reduced ADO analgesia 3 and 5 h after the treatment, while the i.t. doses of 0.1 and 0.3 µg of SCH442416 did not present significant reversion of FBP analgesia (Figure 4D). Moreover, the 1 µg i.pl. treatment with SCH442416 also significantly inhibited the analgesic effect of ADO 3 and 5 h after (Figure 4E). Thus, ADO promotes analgesic effects through activating A1 and A2A receptors, both peripherally and in the spinal cord.

### 2.5. Analgesic Effect of FBP on CCI-Induced Mechanical Hyperalgesia Depends on the Activation of NO/cGMP/PKG/K^+^ATP Signaling Pathway

**Figure 5:**
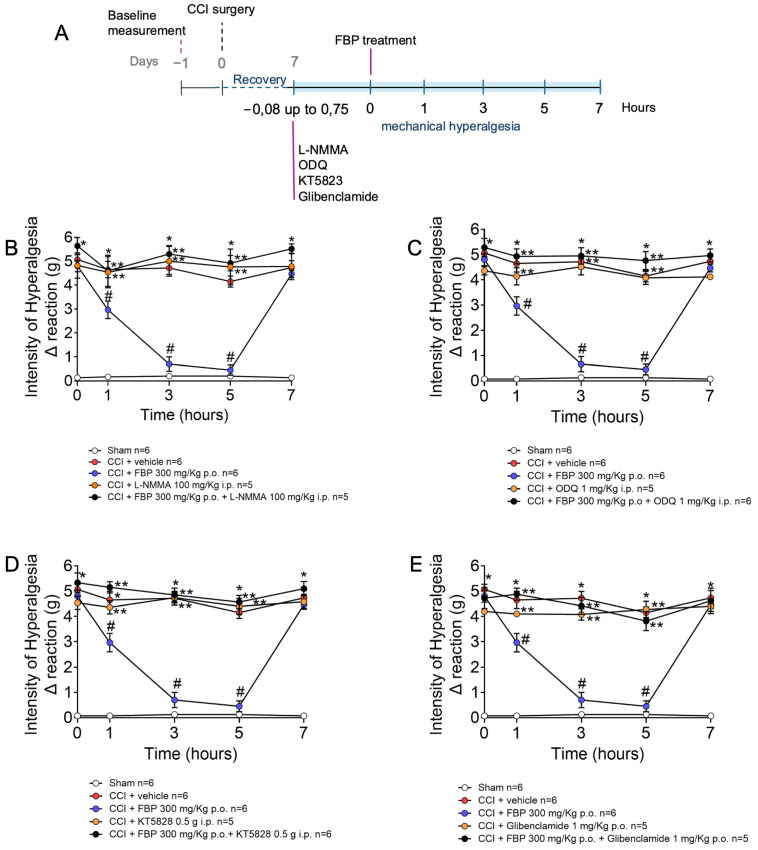
The analgesic effect of FBP depends on the activation of the NO/cGMP/PKG/K^+^ATP signaling pathway. (**A**) Schematic representation of experimental design for this figure. (**B**) Treatment with L-NMMA (100 mg/kg, i.p., 45 min); (**C**) Treatment with ODQ (1 mg/kg, i.p., 30 min); (**D**) Treatment with KT5828 (0.5 µg/animal, i.p., 5 min); and (**E**) glibenclamide (1 mg/kg, p.o., 30 min) before FBP p.o. *n* = 5–6 mice per group per experiment. * *p* < 0.05 CCI vs. sham group; # *p* < 0.05 vs. CCI + vehicle group; ** *p* < 0.05 vs. CCI + FBP 300 mg/kg (Two-way ANOVA followed by Tukey’s post-test).

The A1 receptors are expressed by DRG neurons, and ADO activates A1 receptors to induce analgesia by a mechanism dependent on the nitric oxide/cyclic guanosine monophosphate/protein kinase G/ATP sensitive K channels (NO/cGMP/PKG/K^+^ATP) signaling pathway [31]. Considering that FBP administration increases ADO blood levels by approximately 50%, reaching 1.5 µM in a model of carrageenan inflammatory pain [21], and ADO induces analgesia in CCI neuropathy by activating adenosine A1 and A2A receptors (present data), it was verified whether FBP also activates the NO/cGMP/PKG/K^+^ATP signaling pathway to induce analgesia (Figure 5). At the seventh day after CCI mice were treated with L-NMMA (a nitric oxide synthase inhibitor), ODQ (a soluble guanilate cyclase inhibitor), KT5828 (a PKG inhibitor), or glibenclamide (a K^+^ATP blocker), doses, routes of administration, and time of pre-treatment before FBP treatment are described in Figure 5A. Mechanical hyperalgesia was measured 1–7 h after FBP treatment. CCI induced significant hyperalgesia compared with the sham group, which was significantly inhibited by FBP. On the other hand, the inhibitors L-NMMA, ODQ, KT5828, and glibenclamide diminished the analgesic effect of FBP treatment (Figure 5B–E). Therefore, FBP analgesia depends on activating the NO/cGMP/PKG/K^+^ATP signaling pathway.

### 2.6. FBP Induces NO Production in DRG Neurons In Vitro

**Figure 6:**
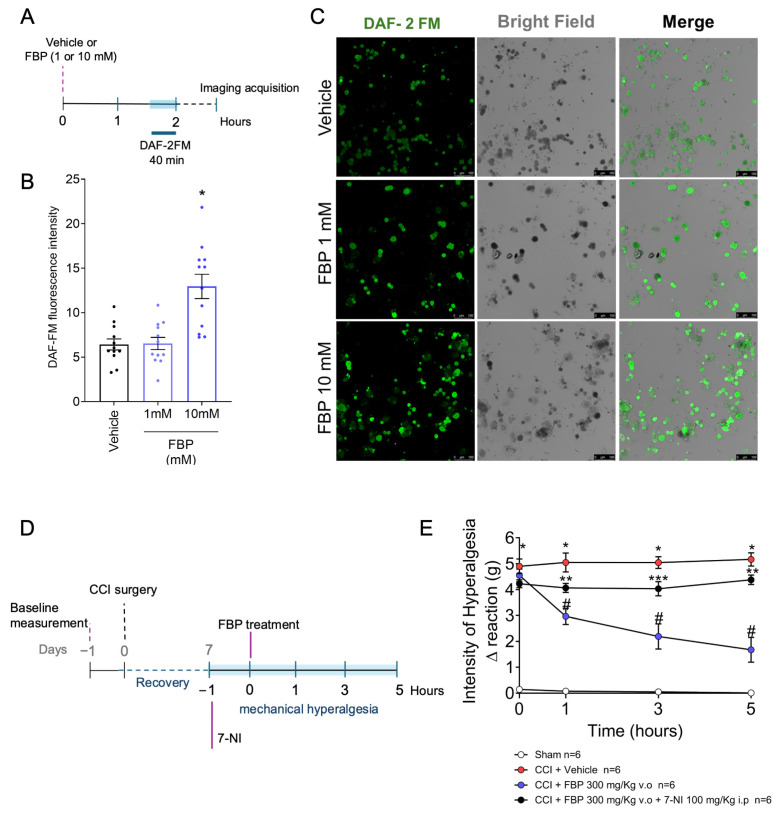
FBP treatment in vitro induces the production of NO in DRG neurons. (**A**) Schematic representation of in vitro experiments for these figures. (**B**) Quantitative analyses of DAF-2 FM fluorescence intensity. (**C**) Representative images from different groups. (**D**) Schematic representation of the in vivo experimental design for this figure. (**E**) Mechanical hyperalgesia of mice treated with FBP (300 mg/hg, p.o.) after 7-NI pretreatment (100 mg/Kg, i.p). *n* = 12 mice per group per experiment for in vitro experiment. For in vivo procedures, *n* = 6 for mechanical hyperalgesia assessment per group per experiment. * *p* < 0.05 vs. vehicle group in vitro or sham group, # *p* < 0.05 vs. CCI + vehicle group, ** *p* < 0.05 vs. CCI + FBP 300 mg/Kg group; *** *p* < 0.05 vs. vehicle group and CCI + FBP 300 mg/kg group. (One-way ANOVA followed by Tukey’s post-test).

We revealed that FBP treatment reduced the mechanical hyperalgesia induced by CCI (Figure 1), and its analgesic effect depends on the activation of the NO/cGMP/PKG/K^+^ATP signaling pathway (Figure 5). Evidence supports that ADO also induces analgesia by activating the NO/cGMP/PKG/K^+^ATP signaling pathway, and that this action would be neuronal because A1 receptors are expressed by DRG neurons [31]. The A2A receptors are also expressed by DRG neurons [48]. In fact, FBP analgesia was reversed by A1 and A2A receptor antagonists administrated i.pl. and i.t., suggesting that a mechanism resembling the primary afferent nociceptor neuron teleantagonism mechanism [47] could be underpinning FBP activity via adenosine receptors. Therefore, as inducing neuronal NO production would be the essential cellular molecular step in this proposed mechanism, it was assessed in the following experiment. We collected DRG of naïve mice and isolated the neurons (Figure 6A). FBP in vitro treatment induced NO production by DRG neurons in a concentration-dependent manner, as determined using the DAF-2FM fluorescent probe (Figure 6B,C). A significant effect was observed with the concentration of 10 mM of FBP (Figure 6B,C). Thus, we demonstrate that FBP induces the neuronal production of NO, which is well-aligned with the results that FBP analgesia is dependent on ADO via A1 and A2A receptors and activating the NO/cGMP/PKG/K^+^ATP signaling pathway, since ADO analgesia is also dependent on the NO/cGMP/PKG/K^+^ATP signaling pathway [31]. To further confirm the participation of neuron-derived NO analgesia induced by FBP treatment, CCI mice were treated on the seventh day after surgery with 7-NI (100 mg/Kg, i.p.) (a selective inhibitor of neuronal nitric oxide synthase [nNOS]) before FBP administration, and mechanical hyperalgesia was assessed (Figure 6D). 7-NI inhibited the anti-hyperalgesic effect of FBP at all observed timepoints (1–5 h) (Figure 6E). Thus, nNOS-derived NO mediated the analgesic activity of FBP in CCI neuropathic pain.

### 2.7. FBP Reduces the CCI Activation of TRPA1^+^ DRG Neurons Population and Reduces the Overt Pain-like Behavior Induced by TRPA1 Agonist in Mice

**Figure 7:**
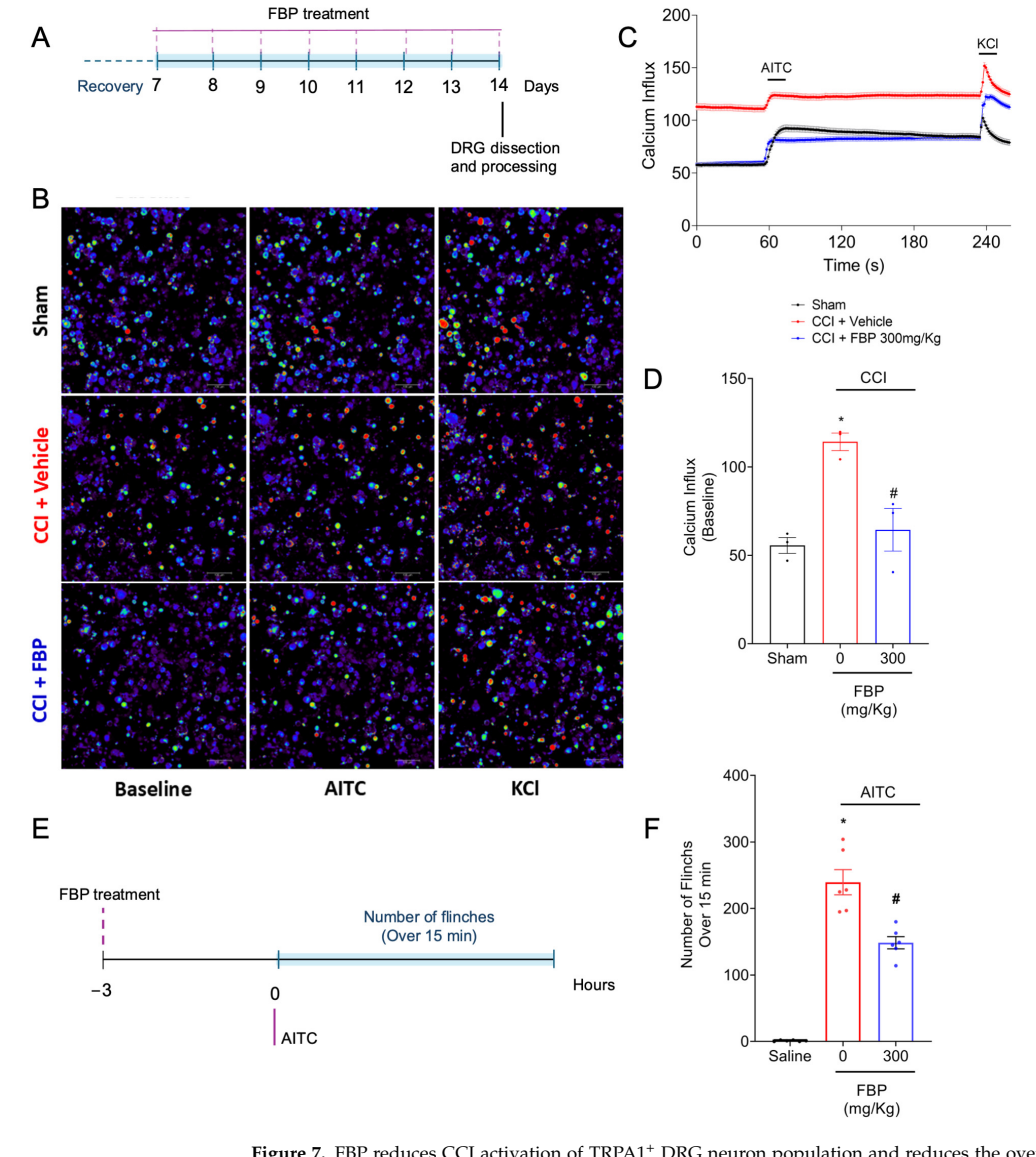
FBP reduces CCI activation of TRPA1^+^ DRG neuron population and reduces the overt pain-like behavior induced by TRPA1 the agonist in mice. (**A**) Scheme of the experimental design of daily treatment of FBP for calcium influx assay. (**B**) Representative images from Sham, CCI + vehicle, and CCI + 300 mg/kg FBP p.o. groups in baseline, AITC (control for identifying TRPA1^+^ neurons) and KCl (control of cell viability) timepoints. (**C**) Traces of calcium influx in baseline, followed by AITC addition and KCl addition. (**D**) Mean values of calcium influx comparing sham, CCI + vehicle, and CCI + FBP. *n* = 3 DRG plates (each plate is a neuronal culture pooled from six mice). (**E**) Scheme of the experimental design of AITC overt pain-like behavior experiment. (**F**) Total number of flinches over 15 min after AITC stimulation in vehicle and FBP-treated groups. *n* = 6 mice per group per experiment. * *p* < 0.05 vs. Sham or saline; # *p* < 0.05 vs. CCI + vehicle or AITC + vehicle (One-way ANOVA followed by Tukey’s post-test).

TRPA1 channels, present in nociceptors, participate in the transduction of noxious stimuli, such as chemical noxious stimuli (e.g., AITC and formalin), into nociceptive signals [3]. In addition, studies have demonstrated their involvement in inflammatory (complete Freund’s adjuvant [CFA] pain model) and neuropathic pain (CCI and spinal nerve ligation models) [49,50]. To investigate if FBP could reduce the activation of TRPA1+ DRG nociceptors population, mice were treated (FBP 300 mg/kg, p.o.) between the 7th and 14th days after CCI surgery. On the 14th day post-CCI, the DRGs were collected and processed for neurons via in vitro culture. The neuronal calcium levels were assessed by applying a fluorescent probe (fluo-4). In order to identify whether the activated neurons were a TRPA1^+^ population, AITC (a TRPA1 agonist) was used after baseline acquisition (Figure 7A). The basal calcium levels of DRG neurons were increased in the CCI group when compared with the sham group. Treatment with FBP reduced the basal calcium levels of the TRPA1^+^ neurons induced by the CCI surgery (Figure 7B–D). TRPA1 channels are permeable to calcium upon activation; thus, calcium represents the activation state of DRG neurons [3]. Considering that the FBP treatment inhibited the basal activation of TRPA1^+^ DRG neurons, we next evaluated the analgesic effect of FBP in an AITC-induced overt pain-like behavior model in vivo. Mice were pretreated with FBP 300 mg/Kg (p.o.) 3 h before the AITC i.pl. injection, and the number of flinches was counted for 15 min (Figure 7E). FBP reduced the number of paw flinches induced by AITC stimuli (Figure 7F). Thus, FBP reduces the CCI activation of TRPA1+ DRG neurons as well as inhibits the nociceptive behavior caused by a TRPA1 agonist, which demonstrates that FBP analgesia depends, at least in part, on inhibiting TRPA1 activation.

## 3. Discussion

FBP exerts anti-inflammatory properties in different inflammatory models in vivo [19,21,51,52] and in vitro [20]. The analgesic action of FBP has been previously demonstrated by our group in inflammatory models of carrageenin- and prostaglandin (PG) E_2_-induced hyperalgesia [21]. Furthermore, FBP can reduce mechanical hyperalgesia in models such as zymosan-induced arthritis (ZIA) and antigen-induced arthritis (AIA) in mice [51]. However, it was unknown whether FBP would inhibit neuropathic pain. Herein, we demonstrate that FBP reduces CCI neuropathic pain in mice. FBP treatment reduced the CCI-induced mechanical hyperalgesia independently of its route of administration (per oral, subcutaneous, intraperitoneal, or intrathecal) and in acute and prolonged treatment protocols. This is the first evidence that FBP is analgesic in neuropathic pain.

The relation between FBP and ADO was first investigated in an ischemia/reperfusion model of the small intestine. This study showed that FBP treatment increases the ATP, AMP, and ADP nucleotides (up to approximately 200 pmol increase/mg of small intestine wet weight) and ADO nucleoside (up to approximately 50 pmol increase/mg of small intestine wet weighy) levels. Furthermore, it was demonstrated that ADO deaminase, an enzyme that converts ADO into an inactive metabolite, reverses the anti-inflammatory actions provided by FBP [53]. Therefore, FBP may act via an increase in the intermediates of glycolysis, which induces an efflux of ATP from cells, resulting in ATP hydrolyzation to ADO by ectonucleotidase action in the extracellular environment, which would then be available to bind to its receptors [21,51,54]. In terms of analgesia, subsequent studies provided evidence that FBP increases ATP and ADO serum levels (up to approximately 500 nM increase) and that treatments with A1 and A2A receptors antagonists prevented FBP analgesia in inflammatory pain [21,51]. This increase in the extracellular levels of ADO by FBP treatment activates adenosine A1 receptor [21], whereas the activation of this receptor triggers the NO/cGMP/PKG/K^+^ATP channel signaling pathway [31]. In the last step of this cascade, ATP-sensitive potassium channels are phosphorylated by protein kinase C (PKC). This action leads to the hyperpolarization of nociceptor neurons by K^+^ efflux. As a consequence of that, the normal depolarization threshold is restored, contrasting with the neuropathic pain in which there is a facilitation of nociceptor neuron depolarization [55,56]. Moreover, it was demonstrated that the increase in serum ADO induced by FBP treatment can elicit antinociception via the adenosine A2A receptor [51]. Furthermore, ADO reduces neuropathic pain by activating the A1 receptor [23,24]. Therefore, considering that FBP reduces inflammatory pain by increasing ADO levels, as well as that ADO reduces neuropathic pain, we hypothesized that FBP could diminish neuropathic pain by increasing ADO levels, allowing for the activation of its receptors.

Here, we further demonstrated that treatment with ADO reduced the mechanical hyperalgesia induced by CCI in mice, corroborating previous studies that showed its analgesic effect in neuropathic pain models [23,24]. Analgesia was observed in per oral and intrathecal vias of administration and in acute and 7-day daily treatment. It is important to highlight that both FBP and ADO reduced mechanical hyperalgesia in a similar profile. ADO receptors A1 and A2A are expressed in neurons of the spinal cord and DRG [21,48,57]. In order to assess the contribution of the spinal cord, DRG, and peripheral A1 and A2A receptors to FBP analgesic activity, we treated mice with A1 receptor antagonist (DPCPX) and A2A receptor antagonist (SCH442416) by intrathecal and intraplantar routes of administration. The central and peripheral delivery of A1 and A2A receptor antagonists inhibited the antinociception of FBP in a dose-dependent manner; moreover, the same profile was observed in ADO-treated mice. These data support the current evidence that FBP exerts its pharmacological antinociceptive action through increasing ADO levels with consequent activation of adenosine A1 and A2A receptors in peripheral and central neurons.

In an inflammatory pain model induced by carrageenin, the antinociceptive effect of FBP was inhibited by treatment with the A1 receptor (DPCPX). In addition, treatment with ADO mimics the analgesia of FBP and this antinociception is prevented by DPCPX in the same manner [21]. These results corroborate our findings and demonstrate that FBP and ADO present similar pharmacological mechanisms for inflammatory and neuropathic pain models. Likewise, A1 receptor antagonists are pronociceptive in inflammatory pain models, such as carrageenin- and formalin-induced pain [31,32,33], as well as in neuropathic pain induced by diabetes and nerve injury [23,37]. Although the exact role of A2A receptors in pain modulation remains controversial, since its activation can exert pronociceptive or antinociceptive effect [41,58,59,60], here, we demonstrated that FBP elicits analgesia in CCI-induced neuropathic pain through A2A receptor activation, which is prevented by SCH442416 in a dose-dependent manner. This result aligns with a previous study that demonstrated that the analgesia promoted by ATL313 (A2A receptor agonist) treatment is abolished by the use of the A2A receptor antagonist (ZM23185) in CCI-induced allodynia. Moreover, the analgesic effect of ATL313 is attributed to the suppression of glial (microglia and astrocyte) activation in the spinal cord [61]. Similarly, in the formalin-induced pain model, the treatment with ZM 241385 inhibited the analgesic action of LASSBio-1359 (an A2A receptor agonist) in the second phase (inflammatory) of the formalin test [59]. The systemic administration of A2A receptors antagonist (ZM241385) reduces the antinociceptive effect of inosine (a metabolite of ADO) in an acetic acid pain model [62]. On the other hand, the antagonism of A2A receptor (DMPX) had no impact on the antinociception elicited by adenosine treatment in diabetes-induced neuropathy [23]. A2A receptor knockout mice present hypoalgesia in acute pain [63]. However, it is also important to highlight that A2A receptor activation can also produce pain, especially when administrated peripherally [58,64], which suggests that A2A may respond differently according to the investigated models.

It was demonstrated that the A1 receptor agonist CPA (N6-cyclopentyladenosine) elicits an analgesic effect in PGE_2_-induced pain through A1 receptor activation, dependent on the NO/cGMP/PKG/K^+^ATP signaling pathway [31]. A2A receptor agonists can also induce analgesia via potassium channel activation [60]. In agreement, the analgesic effect of FBP, which was inhibited by A1 and A2A receptor antagonists, was also abrogated by systemic treatment with inhibitors of the NO/cGMP/PKG/K^+^ATP signaling pathway. Thus, this suggests that FBP, by increasing the ADO levels, would activate the A1 and A2A receptor-dependent activation of the NO/cGMP/PKG/K^+^ATP signaling pathway.

We are presenting the first evidence that FBP can induce analgesia by activating the NO/cGMP/PKG/K^+^ATP signaling pathway, which was further supported by the first demonstration that FBP induces NO production in DRG neurons. Herein, we demonstrated that FBP has direct neuronal effects, since the treatment with FBP in a culture of neurons in vitro increased the NO levels (DAF-2FM fluorescent probe with a 3 nM sensitivity) in these cells in a concentration-dependent manner. Although DRG neurons of naïve mice were used in this in vitro assay, it is noteworthy to mention that the process of dissecting the DRGs can be considered a denervation protocol, since the axons directed to the peripheral tissues, as well as to the spinal cord, are cut. This process does not fully mimic a neuropathic pain state, such as CCI, but at the same time, it is not entirely a naïve condition. Pairing up with FBP induction of NO production by DRG neurons in vitro, the inhibition of nNOS using 7-NI reduced FBP analgesic activity in CCI neuropathic pain. There are examples of drugs in clinical use (e.g., metamizole, diclofenac, and morphine) [65,66,67] and in the pre-clinical phase (e.g., diosmin, kaurenoic acid, nitroxyl, indomethacin, loperamide, and tingenone) [45,56,68,69,70,71], which share the NO/cGMP/PKG/K^+^ATP mechanism of action, highlighting the importance of this signaling pathway in analgesia in inflammatory and neuropathic pain.

Studies have demonstrated that the TRPA1 channels, present in primary afferent neurons, are involved in inflammatory and neuropathic pain [72]. The participation of TRPA1 channels in CCI-induced pain was previously demonstrated, in which the treatment in HC-030031 (TRPA1 antagonist, i.p.) reduced the mechanical allodynia in mice. Furthermore, the i.pl. injection of AITC induced nociception in mice which had previously undergone CCI surgery. The nociceptive response was reduced by HC-030031 pretreatment [50]. The participation of TRPA1 in neuropathic pain was also demonstrated in spinal nerve ligation [49] and chemotherapy-induced neuropathy [73] rodent models. Herein, we demonstrated that CCI induces DRG TRPA1^+^ neuron activation (increase calcium levels), which was inhibited by FBP treatment. Furthermore, FBP treatment reduced the overt pain-like behavior induced by AITC (a TRPA1 agonist). These results suggest that FBP analgesia in the CCI neuropathic pain model involves the inhibition of TRPA1 channels.

Finally, we must mention some limitations of this study and future directions. The present model presents a 100% induction in males and females because the surgical procedure of placing a ligation in the sciatic nerve is sufficient to guarantee such levels of neuropathic pain induction. However, not using female mice is a limitation of the present study considering that evidence supports that there are sex differences regarding pain mechanisms. Female mice present a role of infiltrating T cells in the spinal cord, while male mice present a role of spinal cord glial cells [74,75]. We envisage that developing controlled-release systems can improve the activity of FBP by stably prolonging its release [76]. This study applied a single neuropathic pain mouse model; thus, it would be important to assess whether these results in a sciatic nerve lesion mouse model can be expanded to other types of neuropathic pain with variations in the extent and lesioned nerve, and metabolic induction, such as in diabetic neuropathy and drug-induced neuropathy. We focused on the primary afferent nociceptor neurons because these are the primary lesioned cells in CCI, but secondary effects at other levels may also occur in the spinal cord and brain. Considering that this is the first study demonstrating the analgesic activity and mechanism of action of FBP in neuropathic pain, the present data might open novel venues for FBP application as an analgesic.

## 4. Materials and Methods

### 4.1. Animals

Adult male Swiss mice (25–30 g, 8 weeks old) (total of 621 mice) were obtained from the State University of Londrina and maintained in the department of experimental pathology of the University. The animals were housed in a room with a controlled environment (22 ± 2 °C) under a 12 h light/12 h dark cycle with food and water administered ad libitum, as requested by the animal Ethics Committee of the State University of Londrina (CEUA—No 27348.2010.86, from 2010) and the ethical guidelines of the International Association for the Study of Pain (IASP) [77].

### 4.2. Drugs

The materials used in this study and their sources were: fructose-1,6-biphosphate (FBP, F4757), adenosine (ADO, A9251), NG-monomethyl-L-arginine (L-NMMA 475886), glibenclamide (G0639), and 7-nitroindazole (7-NI, N7778) from Sigma Chemical (St. Louis, MO, USA). SCH442416 (2-(2-Furanyl)-7-[3-(4-methoxyphenyl)propyl]-7H-pyrazolo [4,3-e][1,2,4]triazolo [1,5-c]pyrimidin-5-amine) and 1,3-dipropyl-8-cyclopentylxanthine (DPCPX, 0439) were obtained from Tocris Cookson (Ballwin, MO, USA). KT5823 ((9S,10R,12R)-2,3,9,10,11,12-hexahydro-10-methoxy-2,9-dimethyl-1-oxo-9,12-epoxy-1H-diindolo[1,2,3-fg:3′,2′,1′-kl]pyrrolo[3,4-i][1,6]benzodiazocine-10-carboxylic acid) was obtained from Calbiochem (San Diego, CA, USA). AITC (Allyl isothiocyanate) was obtained from MilliporeSigma (Burlington, MA, USA). FBP, ADO, and AITC were dissolved in saline (0.9%). DPCPX was dissolved in Tween 80 0.5% in saline, Glibenclamide in Tween 80 2% in saline. ODQ and KT5823 were dissolved in DMSO 2% in saline. SCH442416 was dissolved in DMSO 10% in saline. 7-NI was diluted in DMSO 2% and Tween 80 5% in saline.

### 4.3. Experimental Procedures

Baseline mechanical hyperalgesia measures [78] were recorded before the CCI or sham surgeries [79,80] were conducted and were considered the zero-time measurements. Mechanical hyperalgesia evaluation was tested at the 7th day after surgery. FBP or ADO were administrated via the per oral route (p.o.) (30, 100, 300 mg/kg/100 µL) [21,51,79]. The groups were tested for mechanical hyperalgesia again at 1, 3, 5, and 7 h after administration of the drugs. FBP was administrated by p.o., subcutaneous (s.c.), or intraperitoneal (i.p.) (300 mg/kg/100 µL) routes. CCI surgery and behavioral measures were conducted as described in Section 4.4 and Section 4.5. In a different set, between 7 and 14 days after surgery, mice were treated p.o. with FBP, ADO, or vehicle (300 mg/kg/10 µL). The groups were tested for mechanical hyperalgesia again 3 h after administration of the drugs for 7 days. FBP or ADO was administrated intrathecally (3, 10, 30 µg/animal/5 µL). To investigate whether the analgesic effect of FBP was dependent of the adenosine A1 and A2 receptors, the mechanical hyperalgesia was assessed after administration of the A1 and A2 receptor antagonists. DPCPX (adenosine A1 receptor antagonist) was administrated intrathecally (i.t.; 1, 3, 10 µg/animal/5 µL) [80,81] and 30 min later, a dose of FBP or ADO was administrated p.o. (300 mg/kg/100 µL). Afterward, the dose of 10 µg/animal of DPCPX was chosen for intraplantar injection in FBP-treated mice. SCH442416 (adenosine A2A receptor antagonist) was administrated i.t. (0.1, 0.3, and 1 µg/animal/5 µL) and 30 min later, a dose of FBP or adenosine was administrated p.o. (300 mg/kg/100 µL). The dose of 1 µg/animal of SCH442416 was chosen for intraplantar injection in FBP-treated mice. To evaluate whether the analgesia elicited by FBP was through the NO/cGMP/PKG/K^+^ATP signaling pathway, mice were treated via i.p. with L-NMMA (non-selective inhibitor of neuronal nitric oxide synthase) (100 mg/kg/100 µL, 45 min), ODQ (selective inhibitor of the soluble guanylyl cyclase) (1 mg/kg/100 µL, 30 min), or KT5823 (protein kinase G inhibitor) (0.5 µg/animal/100 µL, 5 min), p.o. with glibenclamide (K^+^ATP channel blocker) (1 mg/kg/100 µL, 30 min) [82] or 7-NI (selective inhibitor of neuronal nitric oxide synthase) (10 mg/kg; 1 h) before FBP p.o. treatment (300 mg/Kg/100 µL). In experiments using a single dose, that dose was selected in previous studies [45,83]. To further evaluate the NO participation in the analgesic mechanism of FBP, neurons of naïve mice were cultured and treated in vitro with vehicle or FBP (1 or 10 mM) for 2 h before analyses. To assess the effect of FBP in the activation of TRPA1^+^ neurons, neurons of mice that were treated with FBP treatment (300 mg/Kg p.o.) between the 7th and 14th days after CCI surgery were cultured for the calcium influx assay. To further investigate the effect of FBP in TRPA1 neurons nociception, mice were pretreated with FBP (300 mg/kg, p.o.) 3 h prior to AITC stimuli (1% *v*/*v*, 20 µL i.pl., a TRPA1 agonist) [84], and the number of paw-flinching responses were counted for 15 min.

### 4.4. Chronic Constriction Injury (CCI)

Mice were operated on following Bennett and Xie (1988) methods with minor modifications [85,86]. The animals were anesthetized and the incision was made for access to the right sciatic nerve at the region of the upper femur. One ligature with chromic suture was made in the sciatic nerve without nerve section, only constriction. Bennett and Xie (1988) described this procedure in rats using four ligatures [85]. The increase in mechanical sensitivity in the paw remained significant for approximately 35 days after surgery. In sham animals (false operated), the sciatic nerve was exposed, but not ligated.

### 4.5. Mechanical Hyperalgesia Evaluation

Mechanical hyperalgesia was tested in mice as previously reported [78]. In a quiet room, mice were placed in acrylic cages (12 × 10 × 17 cm) with wire grid floors, 15–30 min before the start of testing. The test consisted of evoking a hind paw flexion reflex with a hand-held force transducer (electronic aesthesiometer; Insight, Ribeirão Preto, SP, Brazil) adapted with a 0.5 mm^2^ polypropylene tip. The investigator was trained to apply the tip perpendicularly to the central area of the hind paw with a gradual increase in pressure. The end point was characterized by the removal of the paw followed by clear flinching movements. After paw withdrawal, the intensity of the pressure was recorded automatically. The value for the response was an averaging of three measurements. The animals were tested before and after chronic sciatic nerve constriction and treatments. The results are expressed by delta (Δ) withdrawal threshold (in g) calculated by subtracting the zero-time mean measurements from the mean measurements.

### 4.6. Overt Pain-like Behaviors

Mice received pretreatment with FBP (300 mg/kg, p.o.) or vehicle 3 h before the stimuli of AITC (1% *v*/*v*, 20 µL i.pl). The total number of paw flinches was counted for 15 min after AITC intraplantar injection. The results are expressed in the total number of flinches [84].

### 4.7. Intracellular Nitric Oxide

To determine the NO production in dorsal root ganglion (DRG neurons), DRG were dissected from naïve Swiss mice and processed as previously described [56]. Neurons were treated with vehicle (Hank’s Balanced Salt Solution (HBSS) (negative control) or FBP (1 or 10 mM) for 2 h and DAF-2FM (Invitrogen, Waltham, MA, USA) diacetate probe was added to the culture and incubated for 40 min in HBSS medium. Plates were washed with HBSS and analyzed via confocal microscopy (TCS SP8, Leica Microsystems, Mannheim, Germany) (20X objective). The NO levels are shown and quantified as the DAF-2FM fluorescence intensity per imaged field.

### 4.8. Calcium Influx Imaging

After surgical recovery, the mice were treated with FBP or vehicle for 7 days. On the 7th day, the DRG neurons were dissected and processed as previously described [87] In brief, DRGs were dissected into Neurobasal-A medium (Life Technologies, Thermo Fisher Scientific, Waltham, MA, USA), dissociated in collagenase A (1 mg·mL^−1^)/dispase II (2.4 U·mL^−1^; RocheApplied Sciences, Indianapolis, IN, USA) in HEPES-buffered saline (MilliporeSigma) up to 10 min at 37 °C and incubated overnight. DRG neuros were then loaded with 1.2 μM of Fluo-4AM probe in Neurobasal-A medium, incubated for 40 min 37 °C, and washed with HBSS. To assess TRPA1 activation, DRG plates were recorded for 5 min, divided into 1 min of initial reading (baseline values), followed by stimulation with AITC 100 µM for 3 min and KCl for 1 min (control, activates all neurons). Calcium flux was imaged in a Confocal Microscope (TCS SP8, Leica Microsystems) (20X objective) and analyzed from the mean fluorescence measured with the Leica Application Suite X (LAS X) Software (Leica Microsystems).

### 4.9. Statistical Analysis

The sample size was determined using G*Power software (version 3.1.9.7, Düsseldorf, Germany) [88]. For the following analyses, GraphPad Prism software version 9 was used. The normality of the data was analyzed by the Shapiro–Wilk test. All data were parametric; thus, they were further analyzed using ANOVA. Two-way ANOVA was used to compare groups and doses at all time points investigated for mechanical hyperalgesia assessment, followed by Tukey’s post hoc test (Figure 1, Figure 2, Figure 3, Figure 4, Figure 5 and Figure 6). One-way ANOVA followed by Tukey’s post hoc test was used to analyze Figure 6 and Figure 7. The results are presented as the mean ± standard error of the mean (SEM) (Supplementary Materials). The specific number of mice per group per experiment is mentioned directly in the figure captions to allow for easier follow up per assay. Statistical differences were considered significant when *p* < 0.05.

## 5. Conclusions

In conclusion, the present data advance by demonstrating, for the first time, the analgesic activity of FBP in a mouse model of neuropathic pain. The FBP analgesic mechanism targets, at least in part, the DRG neurons. Together with the literature data [21,23,37,41,51,64], we concluded that FBP induces an increase in ADO levels, which activates A1 and A2A receptors that trigger neuronal mechanisms dependent on NO to activate the cGMP/PKG/K^+^ATP signaling pathway to cause analgesia. The analgesic effect of FBP also involves targeting the CCI activation of TRPA1^+^ DRG neurons and TRPA1-dependent nociception.

## Data Availability

The data supporting the conclusions of this article will be made available by the authors upon reasonable request.

## Supplementary Materials

The following supporting information can be downloaded at: https://www.mdpi.com/article/10.3390/ph18050660/s1, Table S1 presents the mean, SEM and *n* of each group at indicated time points according to each figure panel.
