# Supplementary material for: Fructose-1,6-Bisphosphate Reduces Chronic Constriction Injury Neuropathic Pain in Mice by Targeting Dorsal Root Ganglia Nociceptive Neuron Activation
Source: Pharmaceuticals (Basel). 2025 Apr 30;18(5):660. doi: 10.3390/ph18050660 (PMC12114996; doi:10.3390/ph18050660)
Supplement: Supplementary file 1 [file pharmaceuticals-18-00660-s001.zip › pharmaceuticals-3214954-supplementary.pdf]

Table S1: Summary of Mean, SEM and *n* of each group at indicated time points according to each figure panel.

Result	Row Statistics			
	**Group**	**Mean**	**SEM**	**n**
**Figure 1A**	Sham			
	0	0,317	0,162	6
	1	0,100	0,052	6
	3	0,367	0,191	6
	5	0,383	0,215	6
	7	0,317	0,162	6
	Vehicle + CCI			
	0	3,633	0,209	6
	1	4,150	0,375	6
	3	3,917	0,207	6
	5	4,200	0,506	6
	7	4,267	0,610	6
	FBP 30 mg/Kg p.o + CCI			
	0	4,167	0,347	6
	1	3,600	0,121	6
	3	3,283	0,224	6
	5	3,583	0,254	6
	7	4,250	0,319	6
	FBP 100 mg/Kg p.o + CCI			
	0	4,350	0,169	6
	1	3,633	0,230	6
	3	1,933	0,472	6
	5	1,483	0,435	6
	7	4,650	0,085	6
	FBP 300 mg/Kg p.o + CCI			
	0	3,400	0,307	6
	1	2,300	0,457	6
	3	0,583	0,291	6
	5	0,600	0,288	6
	7	3,617	0,443	6
	**Group**	**Mean**	**SEM**	**n**
**Figure 1B**	Sham			
	0	0,350	0,193	6
	1	0,200	0,093	6

	3	0,367	0,201	6
	5	0,333	0,158	6
	7	0,667	0,256	6
	Vehicle + CCI			
	0	4,650	0,188	6
	1	5,683	0,266	6
	3	5,333	0,350	6
	5	5,050	0,222	6
	7	5,300	0,447	6
	FBP 30 mg/Kg i.p + CCI			
	0	4,600	0,148	6
	1	4,000	0,294	6
	3	2,200	0,478	6
	5	2,667	0,369	6
	7	4,133	0,186	6
	FBP 100 mg/Kg s.c + CCI			
	0	4,117	0,199	6
	1	4,717	0,353	6
	3	4,400	0,490	6
	5	3,800	0,444	6
	7	3,717	0,282	6
	FBP 300 mg/Kg p.o + CCI			
	0	4,400	0,231	6
	1	3,767	0,173	6
	3	1,500	0,139	6
	5	0,917	0,302	6
	7	4,017	0,298	6

	**Group**	**Mean**	**SEM**	**n**
**Figure 1C**	Sham			
	0	0,150	0,085	6
	1	0,467	0,262	6
	2	0,100	0,052	6
	3	0,400	0,218	6
	4	0,533	0,301	6
	5	0,550	0,292	6
	6	0,333	0,171	6
	7	0,333	0,171	6
	Vehicle + CCI			

	0	4,050	0,154	6
	1	4,033	0,254	6
	2	4,467	0,321	6
	3	4,333	0,364	6
	4	4,467	0,388	6
	5	4,517	0,244	6
	6	4,533	0,334	6
	7	4,533	0,334	6
	FBP 300 mg/Kg p.o + CCI			
	0	4,500	0,169	6
	1	1,283	0,174	6
	2	0,517	0,160	6
	3	1,183	0,266	6
	4	0,467	0,117	6
	5	0,733	0,287	6
	6	1,100	0,299	6
	7	1,100	0,299	6

	**Group**	**Mean**	**SEM**	**n**
**Figure 1D**	Sham			
	0	0,100	0,037	6
	1	0,100	0,052	6
	3	0,200	0,113	6
	5	0,283	0,133	6
	7	0,583	0,279	6
	Vehicle + CCI			
	0	4,400	0,502	5
	1	4,340	0,409	5
	3	4,760	0,617	5
	5	4,420	0,463	5
	7	4,380	0,413	5
	FBP 3 µg i.t + CCI			
	0	4,467	0,287	6
	1	4,020	0,086	5
	3	1,983	0,428	6
	5	2,650	0,209	6
	7	4,750	0,167	6
	FBP 10 µg i.t + CCI			
	0	4,083	0,318	6

	1	2,450	0,258	6
	3	1,000	0,415	6
	5	0,867	0,313	6
	7	4,033	0,278	6
	FBP 30 µg i.t + CCI			
	0	4,067	0,161	6
	1	1,700	0,326	6
	3	0,350	0,186	6
	5	0,100	0,052	6
	7	3,017	0,558	6

	**Group**	**Mean**	**SEM**	**n**
**Figure 2A**	Sham			
	0	0,200	0,103	6
	1	0,283	0,164	6
	3	0,467	0,196	6
	5	0,267	0,133	6
	7	0,317	0,122	6
	Vehicle + CCI			
	0	4,900	0,349	6
	1	4,567	0,396	6
	3	4,867	0,394	6
	5	5,133	0,223	6
	7	4,483	0,237	6
	ADO 30 mg/Kg p.o + CCI			
	0	4,817	0,436	6
	1	2,800	0,288	6
	3	2,717	0,364	6
	5	3,217	0,358	6
	7	4,322	0,258	6
	ADO 100 mg/Kg p.o + CCI			
	0	4,933	0,138	6
	1	2,867	0,272	6
	3	2,350	0,551	6
	5	3,167	0,392	6
	7	4,733	0,403	6
	ADO 300 mg/Kg p.o + CCI			
	0	4,383	0,329	6

	Group	Mean	SEM	n
	1	1,767	0,531	6
	3	1,167	0,531	6
	5	2,000	0,547	6
	7	3,750	0,484	6

	**Group**	**Mean**	**SEM**	**n**
**Figure 2B**	Sham			
	0	0,150	0,085	6
	1	0,457	0,262	6
	2	0,100	0,052	6
	3	0,117	0,065	6
	4	0,550	0,304	6
	5	0,550	0,292	6
	6	0,333	0,171	6
	7	0,333	0,171	6
	Vehicle + CCI			
	0	4,050	0,154	6
	1	4,033	0,254	6
	2	4,467	0,321	6
	3	4,333	0,364	6
	4	4,467	0,388	6
	5	4,517	0,244	6
	6	4,533	0,334	6
	7	4,533	0,334	6
	ADO 300 mg/Kg p.o + CCI			
	0	4,417	0,409	6
	1	1,450	0,440	6
	2	0,867	0,301	6
	3	1,860	0,076	6
	4	1,167	0,293	6
	5	1,367	0,405	6
	6	1,600	0,364	6
	7	1,600	0,364	6

	**Group**	**Mean**	**SEM**	**n**
**Figure 2C**	Sham			
	0	0,139	0,061	6
	1	0,222	0,108	6
	3	0,100	0,052	6
	5	0,122	0,048	6

	7	0,100	0,052	6
	Vehicle + CCI			
	0	4,939	0,206	6
	1	4,789	0,471	6
	3	4,873	0,249	6
	5	4,501	0,304	6
	7	4,634	0,292	6
	ADO 3 µg i.t + CCI			
	0	4,100	0,296	6
	1	3,595	0,612	6
	3	2,838	0,450	6
	5	3,233	0,486	6
	7	4,122	0,281	6
	ADO 10 µg i.t + CCI			
	0	4,283	0,333	6
	1	2,673	0,711	6
	3	2,350	0,853	6
	5	2,867	0,908	6
	7	3,988	0,385	6
	ADO 30 µg i.t + CCI			
	0	3,867	0,364	6
	1	1,733	0,400	6
	3	0,817	0,260	6
	5	0,525	0,239	6
	7	3,200	0,545	6

	**Group**	**Mean**	**SEM**	**n**
**Figure 3A**	Sham			
	0	0,233	0,123	6
	1	0,567	0,312	6
	3	0,267	0,143	6
	5	0,800	0,307	6
	7	0,783	0,380	6
	Vehicle + CCI			
	0	4,667	0,599	6
	1	4,717	0,561	6
	3	5,067	0,654	6
	5	5,400	0,608	6

	Group	Mean	SEM	n
	7	4,833	0,726	6
	FBP 300 mg/Kg p.o + CCI			
	0	3,400	0,307	6
	1	2,300	0,457	6
	3	0,583	0,291	6
	5	0,600	0,288	6
	7	3,617	0,443	6
	FBP 300 mg/Kg p.o + 1 µg DPCPX i.t.+ CCI			
	0	4,840	0,343	5
	1	4,320	0,315	5
	3	2,360	0,262	5
	5	4,660	0,229	5
	7	4,960	0,298	5
	FBP 300 mg/Kg p.o + 3 µg DPCPX i.t.+ CCI			
	0	4,816	0,100	5
	1	4,060	0,548	5
	3	3,260	0,807	5
	5	4,480	0,611	5
	7	4,440	0,308	5
	FBP 300 mg/Kg p.o + 10 µg DPCPX i.t.+ CCI			
	0	5,117	0,281	6
	1	5,050	0,348	6
	3	5,217	0,316	6
	5	5,467	0,293	6
	7	5,233	0,324	6
	**Group**	**Mean**	**SEM**	**n**
**Figure 3B**	Sham			
	0	0,100	0,052	6
	1	0,100	0,052	6
	3	0,100	0,052	6
	5	0,100	0,052	6
	7	0,217	0,108	6
	Vehicle + CCI			
	0	4,933	0,249	6
	1	5,205	0,319	6
	3	4,645	0,430	6
	5	4,750	0,288	6
	7	5,050	0,373	6

	Group	Mean	SEM	n
	FBP 300 mg/Kg p.o.+ CCI			
	0	4,467	0,321	6
	1	3,033	0,371	6
	3	1,067	0,393	6
	5	1,000	0,063	6
	7	4,383	0,366	6
	FBP 300 mg/Kg p.o + 10 µg DPCPX i.pl + CCI			
	0	4,050	0,167	6
	1	3,417	0,320	6
	3	3,717	0,428	6
	5	3,600	0,493	6
	7	4,520	0,307	5

	Group	Mean	SEM	n
**Figure 3C**	Sham			
	0	0,120	0,058	5
	1	0,120	0,058	5
	3	0,120	0,058	5
	5	0,320	0,132	5
	7	0,160	0,093	5
	Vehicle + CCI			
	0	5,140	0,196	5
	1	5,100	0,239	5
	3	5,000	0,277	5
	5	5,328	0,031	5
	7	5,020	0,285	5
	FBP 300 mg/Kg p.o + CCI			
	0	3,886	0,413	5
	1	3,294	0,465	5
	3	0,574	0,246	5
	5	0,740	0,216	5
	7	4,350	0,375	5
	FBP 300 mg/Kg p.o + 0.1 µg SCH442416 i.t.+ CCI			
	0	4,940	0,279	5
	1	4,440	0,308	5
	3	1,160	0,234	5
	5	1,140	0,244	5
	7	4,680	0,314	5
	FBP 300 mg/Kg p.o + 0.3 µg SCH442416 i.t.+ CCI			

	Group	Mean	SEM	n
	0	4,620	0,350	5
	1	4,020	0,431	5
	3	2,720	0,525	5
	5	2,660	0,326	5
	7	4,380	0,482	5
	FBP 300 mg/Kg p.o + 1 µg SCH442416 i.t.+ CCI			
	0	4,900	0,311	5
	1	4,500	0,130	5
	3	4,080	0,185	5
	5	3,520	0,139	5
	7	4,720	0,296	5

	**Group**	**Mean**	**SEM**	**n**
**Figure 3D**	Sham			
	0	0,560	0,333	5
	1	0,560	0,350	5
	3	0,360	0,223	5
	5	0,440	0,277	5
	7	0,600	0,318	5
	Vehicle + CCI			
	0	4,560	0,507	5
	1	4,440	0,565	5
	3	4,800	0,560	5
	5	4,780	0,499	5
	7	4,500	0,466	5
	FBP 300 mg/Kg p.o.+ CCI			
	0	4,520	0,183	5
	1	3,760	0,279	5
	3	0,400	0,130	5
	5	0,640	0,367	5
	7	4,320	0,193	5
	FBP 300 mg/Kg p.o + 1 µg SCH442416 i.pl + CCI			
	0	4,300	0,389	5
	1	4,100	0,400	5
	3	3,680	0,384	5
	5	4,000	0,536	5
	7	4,100	0,365	5

	**Group**	**Mean**	**SEM**	**n**
	Sham			
**Figure 4A**	0	0,255	0,070	6

	1	0,255	0,070	6
	3	0,567	0,255	6
	5	0,417	0,202	6
	7	0,167	0,061	6
	Vehicle + CCI			
	0	4,320	0,426	5
	1	4,540	0,569	5
	3	5,200	0,324	4
	5	4,533	0,455	6
	7	4,860	0,117	5
	ADO 300 mg/Kg p.o + CCI			
	0	4,383	0,329	6
	1	1,767	0,531	6
	3	1,167	0,531	6
	5	2,000	0,547	6
	7	3,750	0,484	6
	ADO 300 mg/Kg p.o + 1 µg DPCPX i.t + CCI			
	0	4,683	0,416	6
	1	2,883	0,547	6
	3	3,500	0,376	4
	5	2,675	0,103	4
	7	4,750	0,331	6
	ADO 300 mg/Kg p.o + 3 µg DPCPX i.t + CCI			
	0	4,783	0,342	6
	1	4,000	0,521	6
	3	4,333	0,395	6
	5	4,033	0,470	6
	7	4,983	0,335	6
	ADO 300 mg/Kg p.o + 10 µg DPCPX i.t + CCI			
	0	4,750	0,292	6
	1	4,433	0,274	6
	3	4,720	0,128	5
	5	4,500	0,288	5
	7	4,817	0,418	6

	**Group**	**Mean**	**SEM**	**n**
**Figure 4B**	Sham			
	0	0,163	0,054	6
	1	0,100	0,045	6
	3	0,150	0,050	6

	5	0,500	0,205	6
	7	0,358	0,124	6
	Vehicle + CCI			
	0	4,933	0,249	6
	1	5,205	0,319	6
	3	4,645	0,430	6
	5	4,750	0,288	6
	7	5,050	0,373	6
	ADO 300 mg/Kg p.o. + CCI			
	0	5,467	0,414	6
	1	3,450	0,254	6
	3	1,783	0,491	6
	5	1,333	0,319	6
	7	5,117	0,406	6
	ADO 300 mg/Kg p.o.+ 10 µg DPCPX i.pl + CCI			
	0	4,583	0,360	6
	1	4,367	0,529	6
	3	3,767	0,516	6
	5	4,450	0,262	6
	7	4,300	0,321	6

	**Group**	**Mean**	**SEM**	**n**
**Figure 4C**	Sham			
	0	0,517	0,244	6
	1	0,142	0,055	6
	3	0,367	0,251	6
	5	0,483	0,299	6
	7	0,179	0,070	6
	Vehicle + CCI			
	0	4,000	0,181	6
	1	4,120	0,229	5
	3	4,400	0,300	5
	5	4,500	0,122	5
	7	4,380	0,248	5
	CCI + ADO 300 mg/Kg p.o.			
	0	4,080	0,248	5
	1	3,460	0,344	5
	3	0,560	0,178	5
	5	0,460	0,186	5
	7	4,020	0,334	5

	Group	Mean	SEM	n
	ADO 300 mg/Kg p.o.+ SCH442416 0.1 µg i.t. + CCI			
	0	4,694	0,252	5
	1	4,334	0,277	5
	3	0,400	0,082	4
	5	0,950	0,096	4
	7	4,820	0,222	5
	ADO 300 mg/Kg p.o.+ SCH442416 0.3 µg i.t. + CCI			
	0	4,280	0,170	6
	1	3,960	0,339	5
	3	1,640	0,480	5
	5	1,800	0,412	6
	7	4,100	0,373	5
	ADO 300 mg/Kg p.o.+ SCH442416 1 µg i.t. + CCI			
	0	4,780	0,378	6
	1	3,960	0,438	5
	3	3,560	0,366	5
	5	2,960	0,390	6
	7	5,120	0,322	5

	**Group**	**Mean**	**SEM**	**n**
**Figure 4D**	Sham			
	0	0,560	0,272	6
	1	0,633	0,308	6
	3	0,433	0,189	6
	5	0,567	0,232	6
	7	0,583	0,281	6
	Vehicle + CCI			
	0	4,560	0,507	5
	1	4,440	0,565	5
	3	4,800	0,560	5
	5	4,780	0,499	5
	7	4,500	0,466	5
	ADO 300 mg/Kg p.o. + CCI			
	0	4,220	0,424	5
	1	3,140	0,435	5
	3	0,075	0,048	4
	5	0,740	0,340	5
	7	4,420	0,567	5
	ADO 300 mg/Kg p.o.+ SCH442416 1 µg i.t. + CCI			
	0	5,000	0,302	5

	1	4,700	0,207	5
	3	4,060	0,326	5
	5	4,340	0,331	5
	7	5,040	0,299	5

	**Group**	**Mean**	**SEM**	**n**
**Figure 5A**	Sham			
	0	0,133	0,033	6
	1	0,167	0,061	6
	3	0,200	0,068	6
	5	0,200	0,068	6
	7	0,133	0,033	6
	Vehicle + CCI			
	0	5,067	0,208	6
	1	4,650	0,343	6
	3	4,717	0,271	6
	5	4,150	0,231	6
	7	4,733	0,288	6
	FBP 300 mg/Kg p.o. + CCI			
	0	4,800	0,238	6
	1	2,967	0,365	6
	3	0,700	0,303	6
	5	0,450	0,214	6
	7	4,467	0,156	6
	L-NMMA 100 mg/Kg i.p. + CCI			
	0	4,820	0,533	5
	1	4,540	0,652	5
	3	5,000	0,622	5
	5	4,760	0,751	5
	7	4,780	0,554	5
	FBP 300 mg/Kg p.o. + L-NMMA 100 mg/Kg i.p. + CCI			
	0	5,640	0,353	5
	1	4,600	0,653	5
	3	5,300	0,352	5
	5	4,920	0,322	5
	7	5,520	0,208	5

	**Group**	**Mean**	**SEM**	**n**
**Figure 5B**	Sham			
	0	0,075	0,030	6
	1	0,075	0,030	6

	3	0,125	0,047	6
	5	0,125	0,047	6
	7	0,075	0,030	6
	Vehicle + CCI			
	0	5,067	0,208	6
	1	4,650	0,343	6
	3	4,717	0,271	6
	5	4,150	0,231	6
	7	4,733	0,288	6
	FBP 300 mg/Kg p.o. + CCI			
	0	4,800	0,238	6
	1	2,967	0,365	6
	3	0,660	0,301	6
	5	0,450	0,214	6
	7	4,467	0,156	6
	ODQ 1 mg/Kg i.p+ CCI.			
	0	4,360	0,163	5
	1	4,140	0,336	5
	3	4,520	0,320	5
	5	4,080	0,256	5
	7	4,120	0,107	5
	FBP 300 mg/Kg p.o. + ODQ 1 mg/Kg i.p. + CCI			
	0	5,283	0,360	6
	1	4,933	0,297	6
	3	4,950	0,327	6
	5	4,767	0,359	6
	7	4,967	0,258	6

	**Group**	**Mean**	**SEM**	**n**
**Figure 5C**	Sham			
	0	0,075	0,030	6
	1	0,075	0,030	6
	3	0,125	0,047	6
	5	0,125	0,047	6
	7	0,075	0,030	6
	Vehicle + CCI			
	0	5,067	0,208	6
	1	4,650	0,343	6
	3	4,717	0,271	6
	5	4,150	0,231	6

	7	4,733	0,288	6
	FBP 300 mg/Kg p.o. + CCI			
	0	4,800	0,238	6
	1	2,967	0,365	6
	3	0,700	0,303	6
	5	0,450	0,214	6
	7	4,467	0,156	6
	KT5828 0.5 µg /Kg i.p. + CCI			
	0	4,540	0,260	5
	1	4,360	0,262	5
	3	4,740	0,214	5
	5	4,400	0,313	5
	7	4,580	0,292	5
	FBP 300 mg/Kg p.o. + KT5828 0.5 µg /Kg i.p. + CCI			
	0	5,333	0,390	6
	1	5,150	0,223	6
	3	4,850	0,272	6
	5	4,567	0,262	6
	7	5,100	0,284	6

	**Group**	**Mean**	**SEM**	**n**
**Figure 5D**	Sham			
	0	0,075	0,030	6
	1	0,075	0,030	6
	3	0,125	0,047	6
	5	0,125	0,047	6
	7	0,075	0,030	6
	Vehicle + CCI			
	0	5,067	0,208	6
	1	4,650	0,343	6
	3	4,717	0,271	6
	5	4,150	0,231	6
	7	4,733	0,288	6
	FBP 300 mg/Kg p.o. + CCI			
	0	4,800	0,238	6
	1	2,967	0,365	6
	3	0,700	0,303	6
	5	0,450	0,214	6
	7	4,467	0,156	6
	Glibenclamide 1mg /Kg p.o. + CCI			

	0	4,200	0,110	5
	1	4,100	0,118	5
	3	4,080	0,222	5
	5	4,280	0,325	5
	7	4,400	0,200	5
	FBP 300 mg/Kg p.o. + glibenclamide 1mg /Kg p.o. + CCI			
	0	4,720	0,414	5
	1	4,880	0,237	5
	3	4,420	0,338	5
	5	3,820	0,377	5
	7	4,620	0,504	5

	**Group**	**Mean**	**SEM**	**n**
**Figure 6A**	Vehicle	6.418	0.621	12
	FBP 1 mM	6.523	0.686	12
	FBP 10 mM	12.949	1.362	12

	**Group**	**Mean**	**SEM**	**n**
**Figure 6E**	Sham			
	0	0,145	0,060	6
	1	0,077	0,071	6
	3	0,050	0,050	6
	5	0,012	0,012	6
	Vehicle + CCI			
	0	4,898	0,281	6
	1	5,045	0,366	6
	3	5,043	0,226	6
	5	5,167	0,255	6
	FBP 300 mg/Kg p.o. + CCI			
	0	4,550	0,242	6
	1	2,967	0,315	6
	3	2,190	0,488	6
	5	1,673	0,481	6

	FBP 300 mg/Kg p.o. + 7-NI 100mg /Kg p.o. + CCI			
	0	4,228	0,151	6
	1	4,063	0,177	6
	3	4,033	0,274	6
	5	4,377	0,184	6
**Figure 7D**	Sham	55,583	4,505	3
	Vehicle + CCI	114,256	4,974	3
	FBP 300 mg/Kg p.o. + CCI	64,513	12,074	3
**Figure 7F**	Saline	1,000	0,365	6
	Vehicle + AITC	239,500	18,834	6
	FBP 300 mg/Kg p.o. + AITC	148,500	9,088	6
